# Improving health related quality of life among rural hypertensive patients through the integrative strategy of health services delivery: a quasi-experimental trial from Chongqing, China

**DOI:** 10.1186/s12939-016-0421-x

**Published:** 2016-08-23

**Authors:** Yudong Miao, Liang Zhang, Vibeke Sparring, Sandeep Sandeep, Wenxi Tang, Xiaowei Sun, Da Feng, Ting Ye

**Affiliations:** 1 School of Medicine and Health Management, Tongji Medical College, Huazhong University of Science and Technology, 13 Hangkong Road, Wuhan, 430030 Hubei Province China; 2 Department of Learning, Informatics, Management and Ethics (LIME) Karolinska Institutet, Stockholm, Sweden

**Keywords:** Hypertension, Health-related quality of life, SF-36, Quasi-experimental trial, Difference-in-differences

## Abstract

**Background:**

Integrative strategy of health services delivery has been proven to be effective in economically developed countries, where the healthcare systems have enough qualified primary care providers. However rural China lacks such providers to act as gatekeeper, besides, Chinese rural hypertensive patients are usually of old age, more likely to be exposed to health risk factors and they experience a greater socio-economic burden. All these Chinese rural setting specific features make the effectiveness of integrative strategy of health services in improving health related quality of life among Chinese rural hypertensive patients uncertain.

**Methods:**

In order to assess the impact of integrative strategy of health services delivery on health related quality of life among Chinese rural hypertensive patients, a two-year quasi-experimental trial was conducted in Chongqing, China. At baseline the sample enrolled 1006 hypertensive patients into intervention group and 420 hypertensive patients into control group. Physicians from village clinics, town hospitals and county hospitals worked collaboratively to deliver multidisciplinary health services for the intervention group, while physicians in the control group provided services without cooperation. The quality of life was studied by SF-36 Scale. Blood pressures were reported by town hospitals. The Difference-in-Differences model was used to estimate the differences in SF-36 score and blood pressure of both groups to assess the impact.

**Results:**

The study showed that at baseline there was no statistical difference in SF-36 scores between both groups. While at follow-up the intervention group scored higher in overall SF-36, Role Physical, Body Pain, Social Functioning and Role Emotional than the control group. The Difference-in-Differences result demonstrated that there were statistical differences in SF-36 total score (*p* = 0.011), Role Physical (*p* = 0.027), Social Functioning (*p* = 0.000), Role Emotional (*p* = 0.002) between both groups. Integrative services delivery improved the score of SF-36 by 4.591 ± 1.794, and also improved the score in domains of Role Physical, Social Functioning and Role Emotional by 8.289 ± 3.753, 9.762 ± 2.019 and 12.534 ± 4.083, respectively.

**Conclusion:**

Patients in the intervention group obtained lower systolic blood pressure and diastolic blood pressure. Integrative strategy of health services delivery improved health related quality of life and blood pressure control among rural Chinese hypertensive patients.

**Trial registration:**

The Ethics Committee of Tongji Medical College, Huazhong University of Science and Technology, ChiCTR-OOR-14005563, Registered on 7 June 2011.

## Background

Studies have shown that patients with clinical hypertension have higher risk of cardiovascular disease, blood pressure-related damage and report lower health-related quality of life (HRQoL). The consequences of this are most devastating in economically developing countries, which are facing problems like social inequalities, economic factors and population growth [1, 2]. Previous studies have pointed out that HRQoL is influenced by physical, psychological, mental, social, and economic circumstances [3]. However, less is studied on how to maintain or promote HRQoL through optimizing health services delivery.

China is a country with about 700 million rural residents whereof 140 million suffer from hypertension. The Chinese rural healthcare system is a 3-level network comprising of county hospitals, township hospitals and village clinics. This huge healthcare system is divided into two parts, the preventive service system and the clinical service system, to provide preventive services and curative services separately for rural residents [4]. With the increasing prevalence of hypertension in rural areas, the Chinese government and rural health care providers came to realize that the patients’ needs were becoming more and more complex and the care approach had to change from individual consultations to multi-professional teamwork.

Experiences from many economically developed countries’ have shown health services integration to be a key approach to delivering health care as well as improving the health outcome of urban community hypertensive patients [5–9]. These healthcare networks have enough qualified physicians (general practitioners and specialty physicians) and residents with some healthcare knowledge. However, in terms of rural healthcare networks in many countries including China, there is a lack of qualified physicians, especially qualified primary care physicians. Moreover, rural hypertensive patients are quite different from their urban counterparts. They are usually of old age, more likely to be exposed to health risk factors and they experience a greater socio-economic burden, which have a great impact on access to health services [10–13]. This suggests that rural hypertensive patients represent a vulnerable population that merits special attention from healthcare providers and systems to deliver appropriate healthcare services [14]. Therefore since China’s 2009 health care reform, the government had set a series of health policy trying to integrate the health services delivered by physicians across the 3-level network to deal with chronic diseases. However how to implement the integrative strategy of health services delivery in rural China and whether it was effective among rural hypertensive patients were still uncertain.

In this study, we conducted a two-year quasi-experimental trial in collaboration with Qianjiang District Health Board of Chongqing Municipality, China, to estimate whether or not the integrative strategy of health services delivery can improve HRQoL among rural hypertensive patients. To our knowledge, this study is the first of this kind. Similar interventions have not been piloted in other settings of China and the result will provide crucial information (the design, execution and assessment of integrative care delivered by inter-agency physicians) to guide the management and treatment of patients with hypertension for rural China, as well as many other economically developing countries.

## Methods

### Sampling and intervention assignment

**Fig. 1:**
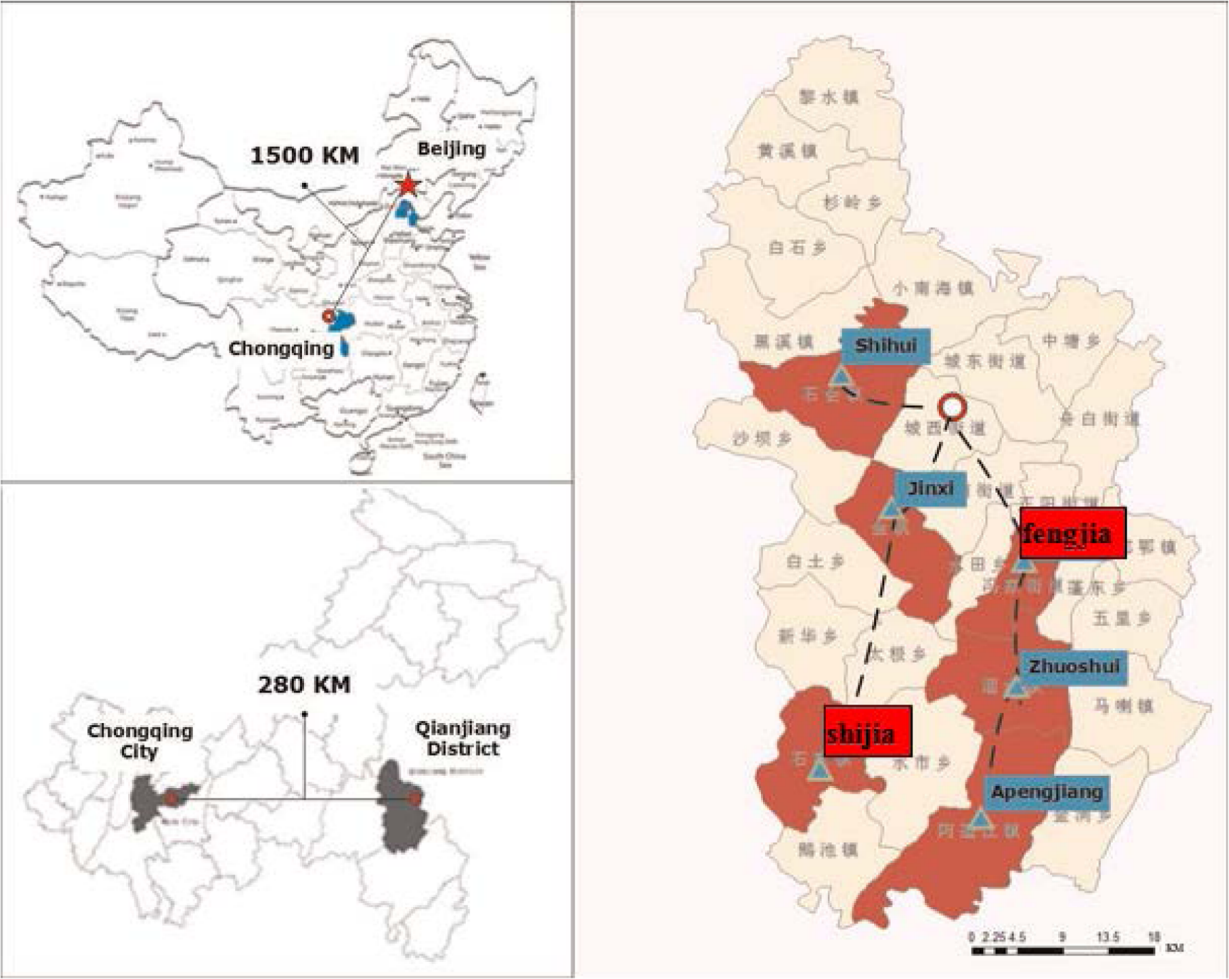
Showed the location of the six towns enrolled into this quasi-experimental trial

This quasi-experimental trial was conducted for two years from July, 2012 to August, 2014 focusing on the effects of the integrative health services on HRQoL in rural hypertensive patients as sub-project of the research Study on the Efficiency and Effectiveness of the Integrated Health Care Services in Rural China, funded by China Medical Board (CMB). The study randomly selected six towns (Apengjiang, Jinxi, Zhuoshui, Shihui, Fengjia, Shijia) out of all 30 towns in Qianjiang district of Chongqing Municipality of China (see Fig. 1). In each sampled town, all registered patients with hypertension were enrolled into the trial. Three towns (Apengjiang, Jinxi, Zhuoshui, Shihui; *n* = 1006) were assigned to intervention group and the remaining three towns (Fengjia, Shijia; *n* = 420) were assigned to control group. This assignment was decided according to the appeals of the directors of chronic non-communicable diseases management directors of each town. All valid patients were surveyed for studying HRQoL at baseline (*n* = 1426, July to August of 2012). Two years after the intervention, the HRQoL of the respondents were surveyed once again at the follow-up (*n* = 1083, July to August of 2014). Both surveys at baseline and follow-up were conducted from July to August to minimize the seasonal variation.

### Participants’ inclusion and exclusion criteria

Included in the study were participants who: 1) were diagnosed with hypertension; 2) were local rural residents aged 18 or above and willing to participate and who signed the informed consent; 3) had their name in the chronic disease management information system of township hospital and had received hypertension treatment by township hospital doctors; 4) were able to complete the surveys by SF-36 Scale independently or in assistance of another person. Patients who 1) were not permanent residents of the sampled towns; 2) Refused or were unable to fully participate in the entire intervention; 3) Had a life expectancy of less than 2 years before study participation due to severe disease confirmed by county hospital physicians or old age, were excluded from the study.

### Measure of HRQoL

The Medical Outcome Study Short-Form 36-Item Health Survey (SF-36 Scale) was used to assess HRQoL and the differences in scores between the intervention group and the control group represented the effect of the intervention. The SF-36 scale was developed by the RAND Corporation’s Health Insurance Experiment in the United States [15], and the Chinese version of the SF-36 scale used in this study has been widely used for evaluations of HRQoL, clinical trials and health policy in China, and proven to have good validity and reliability [16, 17].

**Fig. 2:**
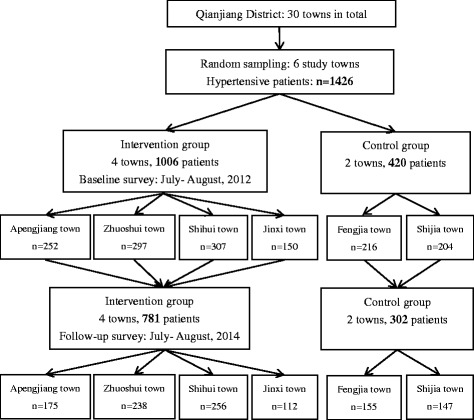
Showed the study flow chart outlining the recruitment, allocation to different groups and the field surveys on the health related quality of life in rural hypertensive patients

The SF-36 scale consists of 36 items divided into 8 dimensions: physical functioning (PF), role physical (RP), bodily pain (BP), general health (GH), vitality (VT), social functioning (SF), role emotional (RE) and mental health (MH). Each dimension scores from 0 (poorest health) to 100 (optimal health), and the SF-36 scores an average of the 8 dimensions, namely the score of SF 36 = (PF+ RP+ BP+ GH+ VT+ SF+ RE+ MH)/8. The score of SF-36 represented the overall HRQoL, the higher the score, the better the HRQoL. The study design and the flow of participants are shown in Fig. 2.

### Intervention

The two-year interventional study was designed to provide integrative health services for a rural population with hypertension. Physicians in village clinics, township hospitals and county hospitals worked collaboratively to implement the intervention. The intervention package mainly included two parts: integration of preventive-curative services delivery and cooperation among village-town-county physicians.

Since China’s rural health system lacks general practitioners to act as gatekeeper, village clinic physicians play the major role in ensuring the implication of the integrative strategy due to their continued and interactive relationship with patients. Village clinic physicians worked under the guidance of township hospital physicians to monitor the blood pressure once per month and the BMI twice per year. Patients whose blood pressure and BMI control worsened were offered more visits by telephone calls to intensify disease control. Village clinic physicians followed patients proactively to support adherence to drugs, using motivational coaching methods [7, 18, 19]. Moreover, they provided goal-oriented health education mainly focused on smoking cessation, moderate drinking, light and healthy diet, regular exercise and mastering skills to alleviate negative emotions according to individualized lifestyle and health education scheme made by township hospital physicians. This scheme also emphasized village clinic physicians’ structured visits every month to instruct patients with hypertension to comply with the health education contents and to take blood pressure drugs regularly to achieve good health outcomes.

Because of the general lack of health awareness, Chinese rural hypertensive patients usually did not visit the doctor when they had hypertension symptoms. According to China Health Statistics Annual Report, the treatment rate in rural China was only 17.4 % [20]. Therefore, the village clinic physicians in this study were urged to learn about their patients’ health conditions, lifestyle, drug use and treatment by telephone every two weeks, and to give guidance on timely treatment to the hypertensive patients who needed clinical services. The health status of the patients and the timely treatment guidance were recorded by the village clinic physicians and reported to township hospital physicians.

Township hospital physicians acted as integrators for a combination of preventive-curative services and a further patient movement within county-township health system. Township hospital physicians made individualized lifestyle and health education schemes according to the patient reports from the village clinic physicians, and gave advice to village clinic physicians on the adjustment of the health education carried out in previous stages in accordance with patients’ health condition and disease progress. Township hospital physicians conducted medical examinations 2 times per year (1st in March or April, and 2nd in September or October) to screen for complications and established health records for patients with hypertension. They provided curative and rehabilitative care after admissions and for patients with severe hypertension or complications, township hospital physicians implemented a township-to-county referral with a referral request form recognized by county hospital to ensure patients’ timely access to treatment.

**Fig. 3:**
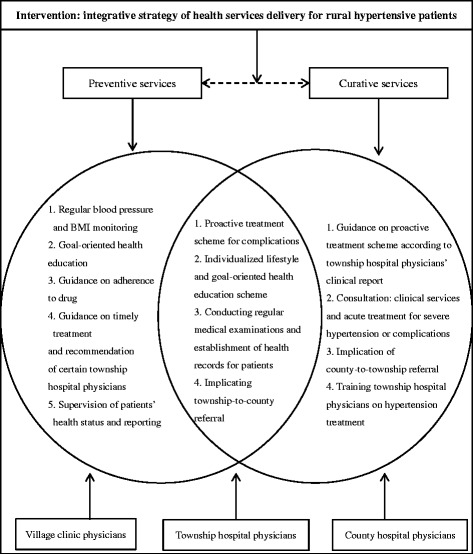
Showed the integrative strategy of health services delivery: the main inclusive elements of the intervention contributed collaboratively by physicians in the 3-level healthcare system

County hospital physicians played a leadership role in the delivery of the multi-disciplinary care. They gave guidance on the adjustment of the proactive treatment scheme made by township hospital physicians according to their clinical reports. A consultation regulation was set up in the county hospital to provide clinical services or acute treatment for referral patients with severe hypertension or complications from the township hospitals. County hospital physicians implemented a county-to-township referral when patients entered rehabilitation or a stable condition. Moreover, as township hospital physicians lacked treatment skills of hypertension or knowledge of complications generally in China, county hospital physicians in this intervention were urged to train township hospital physicians in hypertension or complications treatment. Every month, the county doctors would go to township hospitals to give lectures to village and town physicians on how to diagnose and treat patients with hypertension or diabetes, to review the last month discharged patients (or else they had to travel long to have their own) and to have case discussions with the local staff. The main measures of the intervention package were shown in Fig. 3.

Preventive or curative services in the usual care group were still delivered by single physician in the village-town-county healthcare system independently, and there was no integrative strategy implicated.

### Study oversight

This study set up a Project Quality Supervision Committee composed of a health management expert, a Qianjiang District Health Board official, a county hospital doctor, a township hospital doctor and a village doctor to guide and monitor the implementation of the intervention.

### Statistical analysis

Quantitative data at baseline and follow-up were presented as means with standard deviations, distributions of categorical data were presented as absolute frequencies and percentages [21]. *T*-test was conducted to test the differences between means and repeated measures ANOVA was used to compare proportions and drop rate and to estimate whether the compositions of the intervention group and control group were comparable. Considering the missing visits of observed patients with hypertension in the two-year quasi-experimental trial, this study used the fixed effect estimation method of Difference-in-Differences (DID) to estimate the differences of SF-36 scores and blood pressures between the intervention group and control group to assess the effects. The DID analysis is a quasi-experimental method which had been widely used to evaluate project outcomes, mostly in public health projects. The method pretends to capture the effects related with some treatment or event through time, between a control group and a treatment group [22]. For the observed individual *i*, the basic settings for DID model were:

$$ {y}_i = {\beta}_0 + {\beta}_1\kern0.5em \cdotp \kern0.5em tim{e}_i + \gamma \kern0.5em \cdotp \kern0.5em  grou{p}_i + \delta \kern0.5em \cdotp \kern0.5em  grou{p}_i\cdotp \kern0.5em tim{e}_i+{\varepsilon}_i $$

The estimated coefficients in this linear regression had the following interpretation:

$\overset{\wedge }{\upbeta 0}$: Mean outcome for the control group at baseline.

$\overset{\wedge }{\upbeta 0}\kern0.5em +\kern0.5em \overset{\wedge }{\upbeta 1}$: Mean outcome for the control group at follow-up.

$\overset{\wedge }{\upgamma}$: The single difference between treatment and control groups at baseline.

$\overset{\wedge }{\upbeta 0}\kern0.5em +\kern0.5em \overset{\wedge }{\upgamma}$: Mean outcome for the treatment group at baseline.

$\overset{\wedge }{\upbeta 0}\kern0.5em +\kern0.5em \overset{\wedge }{\upbeta 1}\kern0.5em +\kern0.5em \overset{\wedge }{\upgamma}\kern0.5em +\kern0.5em \overset{\wedge }{\updelta}$: Mean outcome for the treatment group at follow-up.

$\overset{\wedge }{\updelta}$: The DID or impact of the intervention.

*ε*_*i*_: The random error.

A value of *P* < 0.05 was considered to be statistically significant. Questionnaire data entry was conducted by using Epidata version 3.0 (http://www.epidata.dk/download.php) and data were analyzed using STATA statistical software version 11.0(http://www.stata.com/stata11/).

## Results

### Study group characteristics

Patients in both groups are similar at baseline and follow-up. Table 1 shows the frequency of patients in each town. Of the 1426 patients enrolled (*n* = 1006 intervention group and *n* = 420 control group), 75.9 % completed the two-year intervention and the SF-36 scale assessments (*n* = 781, 77.5 % in the intervention group and *n* = 302, 71.9 % in the control group). The patients’ socio-demographic characteristics and duration of hypertension are shown in Table 2. The results show no significant differences between the intervention group and the control group, and thus avoided the influence of these confounding factors.

**Table 1:** Composition of the intervention group and control group

Group	Town	Baseline		Follow-up		*P*
		Frequency	Percentage (%)	Frequency	Percentage (%)	
Intervention group		1006	70.5	781	72.1	*P > 0.05*
	Apengjiang	252	17.7	175	16.2	*P > 0.05*
	Zhuoshui	297	20.8	238	22.0	*P > 0.05*
	Shihui	307	21.5	256	23.7	*P > 0.05*
	Jinxi	150	10.5	112	10.3	*P > 0.05*
Control group		420	29.5	302	27.9	*P > 0.05*
	Fengjia	216	15.1	155	14.3	*P > 0.05*
	Shijia	204	14.3	147	13.6	*P > 0.05*
Total		1426	100.0	1083	100.0	*P > 0.05*

**Table 2:** Socio-demographic characteristics of the patients surveyed by SF-36 scale at baseline and follow-up

Variables		Baseline			Follow-up		
		Intervention group (*N* = 1006)	Control group (*N* = 420)	*p*	Intervention group (*N* = 781)	Control group (*N* = 302)	*p*
Sex (%)				*p > 0.05*			*p > 0.05*
	Male	45.2	44.0		44.9	55.1	
	Female	54.8	56.0		44.7	55.3	
Age(year; $\overline{\mathrm{x}}\pm \mathrm{s}$)		66.3 ± 10.7	65.8 ± 11.5	*p > 0.05*	68.20 ± 11.0	67.79 ± 11.5	*p > 0.05*
Education (%)				*p > 0.05*			*p > 0.05*
	Illiteracy	35.0	39.5		17.4	17.2	
	Primary school	47.3	44.5		29.4	36.1	
	Middle school or higher	17.2	16.0		53.2	46.7	
Occupation (%)				*p > 0.05*			*p > 0.05*
	Farmer	94.1	96.2		93.2	94.7	
	Non-farmer	5.9	3.8		6.8	5.3	
Duration of hypertension (year; $\overline{\mathrm{x}}\pm \mathrm{s}$)		3.4 ± 4.6	3.8 ± 4.6	*p > 0.05*	5.4 ± 4.6	5.8 ± 4.6	*p > 0.05*

### Drop out rate at follow-up

A total of 343 (24.1 %) individuals had dropped out at follow-up, where of 225(24.1 %) were in intervention group and 118 (23.2 %) in control group. The drop out rate between both groups had no statistical difference (*P* > 0.05). In the intervention group, reasons for drop out were long-term working in city (58.5 %), moving to city (21.8 %), lack of time to respond in the follow-up field survey (16.5 %), declined to respond in the follow-up field survey without informing any reason (3.2 %). In the control group, 169 drop outs were caused by long-term working in city (52.7 %), moving to city (27.7 %), lacking time to respond in the follow-up field survey (9.5 %), declined to respond in the follow-up field survey without informing any reason (10.1 %).

### The impact of integrative strategy of health services delivery on HRQoL

Table 3 shows the effects of integrative strategy on HRQoL. At baseline, there were no significant differences in SF-36 overall score (0 ~ 100) between both groups, while the intervention group scored lower in GH, SF and RE than the control group by 3.546 ± 1.499, 6.091 ± 1.314 and 5.956 ± 2.657. At follow-up, the intervention group scored higher in overall SF-36, RP, BP, SF and RE than the control group by 8.57 ± 2.85, 3.613 ± 1.625, 3.672 ± 1.532 and 6.578 ± 3.099. There were no statistically significant differences in BP which was the case at baseline. The DID result showed statistically significant differences in SF-36 total score (*P* = 0.011), RP (*P* = 0.027), SF (*P* = 0.000), and RE (*P* = 0.002) between the intervention and control group. Integrative health services delivery improved the score of SF-36 by 4.591 ± 1.794, and the intervention also improved the score in dimensions of RP, SF and RE by 8.289 ± 3.753, 9.762 ± 2.019 and 12.534 ± 4.083, respectively. The DID result also showed that our intervention also improved PF, BP, VT, and MH in the follow-up assessment, but there were no statistical differences (*P* > 0.05). GH score lowered (-1.253) during the intervention, but also there was no statistical difference (*P* > 0.05).

**Table 3:** Health related quality of life estimated by DID model with STATA

Outcome Variable (y)	Baseline			Follow-up			
	Control $\left({\widehat{\upbeta}}_0\right)$	Intervention $\left({\widehat{\upbeta}}_0+\widehat{\upgamma}\right)$	Diff (BL) $\left(\widehat{\upgamma}\right)$	Control $\left({\widehat{\upbeta}}_0+{\widehat{\upbeta}}_1\right)$	Intervention $\left({\widehat{\upbeta}}_0+{\widehat{\upbeta}}_1+\widehat{\upgamma}+\widehat{\updelta}\right)$	Diff (FU) $\left(\widehat{\upgamma}+\widehat{\updelta}\right)$	DIFF-IN-DIFF $\left(\widehat{\updelta}\right)$
SF-36	51.426	49.588	−1.839	53.93	56.683	2.753	4.591
*S.E.*	0.98	0.634	1.167	1.156	0.719	1.362	1.794
*p*			0.115			0.043	0.011
PF	63.179	64.622	1.443	63.974	66.994	3.02	1.577
*S.E.*	1.303	0.842	1.551	1.536	0.956	1.81	2.384
*p*			0.352			0.095	0.508
RP	27.381	27.662	0.281	32.616	41.186	8.57	8.289
*S.E.*	2.052	1.326	2.443	2.419	1.505	2.85	3.753
*p*			0.909			0.003	0.027
BP	56.376	56.388	0.012	60.079	63.692	3.613	3.601
*S.E.*	1.17	0.756	1.393	1.38	0.858	1.625	2.14
*p*			0.993			0.026	0.093
GH	41.629	38.083	−3.546	44.298	43.045	−1.253	2.293
*S.E.*	1.259	0.814	1.499	1.485	0.924	1.749	2.303
*p*			0.018			0.474	0.32
VT	52.25	52.214	−0.036	56.995	58.686	1.691	1.727
*S.E.*	0.924	0.597	1.1	1.089	0.678	1.283	1.69
*p*			0.974			0.188	0.307
SF	71.339	65.249	−6.091	70.447	74.119	3.672	9.762
*S.E.*	1.103	0.713	1.314	1.301	0.81	1.532	2.019
*p*			0.000			0.017	0.000
RE	44.762	38.806	−5.956	49.448	56.026	6.578	12.534
*S.E.*	2.232	1.443	2.657	2.632	1.637	3.099	4.083
*p*			0.025			0.034	0.002
MH	54.495	53.68	−0.816	60.149	61.346	1.197	2.013
*S.E.*	0.874	0.565	1.041	1.031	0.641	1.214	1.599
*p*			0.433			0.324	0.208

* Means and Standard Errors were estimated by linear regression.

### The impact of integrative strategy of health services delivery on blood pressure

At baseline, there were no significant differences in blood pressure in neither groups, while at follow-up, the intervention group mean systolic blood pressure (SBP) was 131.55 ± 9.17 mmHg and mean diastolic blood pressure (DBP) was 90.85 ± 9.89 mmHg (see Table 4). The DID modeling results indicated that the intervention lowered the SBP and DBP by -5.62 ± 16.49 mmHg and -5.43 ± 15.07 mmHg respectively.

**Table 4:** Blood pressure (mm Hg) estimated by DID model with STATA

Outcome Variable (y)	Baseline			Follow-up			
	Control $\left({\widehat{\upbeta}}_0\right)$	Intervention $\left({\widehat{\upbeta}}_0+\widehat{\upgamma}\right)$	Diff (BL) $\left(\widehat{\upgamma}\right)$	Control $\left({\widehat{\upbeta}}_0+{\widehat{\upbeta}}_1\right)$	Intervention $\left({\widehat{\upbeta}}_0+{\widehat{\upbeta}}_1+\widehat{\upgamma}+\widehat{\updelta}\right)$	Diff (FU) $\left(\widehat{\upgamma}+\widehat{\updelta}\right)$	DIFF-IN-DIFF $\left(\widehat{\updelta}\right)$
SBP	137.15	139.08	1.93	135.24	131.55	−3.69	−5.62
*S.E.*	10.13	8.64	12.76	8.42	9.17	11.02	16.49
*p*			0.205			0.047	0.019
DBP	97.28	96.44	−0.84	97.12	90.85	−6.27	−5.43
*S.E.*	11.09	10.52	14.41	11.25	9.89	13.66	15.07
*p*			0.557			0.013	0.028

* Means and Standard Errors were estimated by linear regression.

## Discussion

HRQoL measured perceived health and the actual functions were the main determinants [23]. The DID model in this study showed that our intervention improved the overall score of SF-36, as well as the domains of RP, SF and RE. One possible reason was that during the trial, physicians in the intervention group gave more guidance to patients on timely seeking clinical treatment. Meanwhile, the relationship between physicians and patients became closer and patients may have been able to obtain the friendship and health care they desired from the physicians, which contributed to the improved HRQoL. The other possible reason was that our study helped to control blood pressure, which is critical for hypertensive patients [24, 25]. The DID method took into account the differences between HRQoL and blood pressure of both groups prior to the implementation of the intervention, which ensured our results more reliable.

The Chinese chronic non-communicable diseases population management has long been plagued by the discordance between preventive and curative care providers. In the treatment of hypertensive patients, there were two widely recognized strategies - the ‘prevention’ strategy and the ‘clinical treatment’ strategy. Both strategies were considered to be effective by the government and healthcare providers, and therefore public health services and clinical services were provided separately [26]. The lack of necessary interface between both strategies finally resulted in a fragmented healthcare delivery in chronic disease prevention [27]. Discordance in health service delivery is a problem in all health systems, and the major challenge is to strengthen integration in order to enhance efficiency and health outcomes [28]. Although China in recent years has been committed to promoting the vertical integration of medical institutions to provide continuous clinical services, hypertensive patients in rural China, like in rural areas of many other economically developing countries, still experience poor access to healthcare and poor health outcomes compared to metropolitan populations, due to the lack of health consciousness and good habits of seeking health services [29, 30]. One of the major challenges is to find strategies for strengthening hypertension management and guiding patients to rational clinical treatment [31]. The design of the intervention in the present study described a clear path to the integration of hypertension management and clinical treatment through the collaboration among inter-agency physicians in the 3-level healthcare system in rural areas, which provided a good strategy for hypertension prevention and treatment.

Another very worrisome problem is that many physicians working in the Chinese rural healthcare system, especially physicians in the preventive care system are not qualified physicians by western standards but are healthcare workers with limited medical training, basic equipment and a restricted pharmacopeia [32, 33]. This called for the collaboration between providers in the 3-level network to increase the overall capacity of delivering health services. In this study, collaboration among multi-disciplinary and cross-level physicians was essential to the intervention, which was also very important in health care reform [34]. The positive results of this study indicated that even though there were not enough qualified primary care providers in rural China, this integrative strategy could also be implemented through the collaboration between rural healthcare physicians and thereby improve the health outcomes of rural hypertensive patients. This may enlighten rural China and many other economically developing countries to put forward healthcare providers’ integration policy to enhance health services delivery.

Quite a few previous studies reported the impact of integrative strategy of health services delivery on HRQoL. For example, in one quasi-experimental trial, Wilhelmina et al. conducted a three-month integrated care program to evaluate the effects of the integrated care delivered by general practitioners on HRQoL in frail elderly patients. The results showed that the HRQoL in frail elderly patients improved due to the short-term effects of integrated care [35]. Compared with our study, general practitioners in the three-month integrated care program played role as providers, whereas the Chinese 3-level healthcare network lacked qualified general practitioners, and it is therefore uncertain whether the intervention in this the three-month integrated care program worked or not in rural China. Our study process lasted two years and demonstrated the positive effects, which broadened the research scope. Another study carried out in Germany healthcare settings found that integrated inpatient health care program did not seem to improve the patients’ HRQoL and health status, but positive effects on patient satisfaction and the efficiency of hospital care could be detected. The authors proposed that future research ought to check the advantages and/or disadvantages of IHC for other disease groups, and should, if possible, use a prospective study design as well as integrate clinical parameters [36]. The present 2-year quasi-experimental study was prospective in study design and data analysis. From this perspective, our study was of particular significance in demonstrating the effectiveness of integrative strategy of health services delivery on HRQoL within rural Chinese healthcare settings, and maybe other healthcare systems lacking qualified primary care physicians.

This study has limitations that should be pointed out. In this 2 year quasi-experimental study based on hypertensive population, the 24.1 % drop out rate may be higher than other studies. We foresaw this and all program designers debated for several rounds, and finally we enrolled all valid sampling patients strictly according to the inclusion and exclusion criteria so as to reduce the drop outs and its impact on the results. Considering the fact that the drop outs were random, we think the loss was inconceivable and the statistical analysis results were reasonable. Moreover, the DID method itself had avoided the confounding effects caused by the drop outs due to the time effect. Furthermore, since rural China is economically developing and has more limited healthcare resources compared to urban areas [32, 37], the cost-effectiveness of the integrative health services delivery strategy must be taken into consideration in hypertension management. Therefore, future studies should focus on the cost and the efficiency of the integration to determine whether this service delivery model is proper and sustainable.

## Conclusion

In conclusion, integrative strategy of health services delivery in this quasi-experimental trial improved the HRQoL among rural hypertensive patients. Future chronic non-communicable diseases management in rural China, and many other economically developing countries lacking enough qualified primary care providers, should put emphasis on the collaboration among physicians and on the integrative health services delivery.

### Summary table

1. What is known about this topic:
  Integrative strategy of health services delivery has been proven to be effective in economically developed countries, where the healthcare systems have enough general practitioners or qualified physicians.
  Hypertensive patients in previous studies were mainly sampled from urban areas. They usually had some basic healthcare knowledge that the rural patients lacked.
2. What this study adds:
  This study is a quasi-experimental trial conducted in rural China related to the assessment of the impact of health service delivery integration on health related quality of life.
  This study propounded an integrative strategy of health services delivery suitable for rural Chinese hypertensive patients.
  This study used the DID method to assess the intervention effects.
  This study focused on rural hypertensive patients who represent a vulnerable population.

## Abbreviations

HRQoL: Health related quality of life
DID: Difference-in-differences
SF-36: The medical outcome study short-form 36-item health survey
PF: Physical functioning
RP: Role physical
BP: Bodily pain
GH: General health
VT: Vitality
SF: Social functioning
RE: Role emotional
SBP: Systolic blood pressure
DBP: Diastolic blood pressure
